# Development of a Generalist Competency Framework for Medical Education Leaders in Longitudinal Integrated Curricula (LILIC)

**DOI:** 10.1007/s40670-024-02185-8

**Published:** 2024-11-08

**Authors:** Charles A. Gullo, Youngjin Cho, John L. Szarek, Gabi N. Waite, Kelly M. Quesnelle, Amy Prunuske

**Affiliations:** 1 Gullo Consulting, LLC, Fort Worth, TX USA; 2 Department of Medical Education, Geisinger Commonwealth School of Medicine, Geisinger College of Health Sciences, Scranton, PA USA; 3 Department of Medical Education, Geisinger Commonwealth School of Medicine, Scranton, PA USA; 4 Department of Biomedical Sciences, University of South Carolina School of Medicine Greenville, Greenville, SC USA; 5 Medical College of Wisconsin - Central Wisconsin, Wausau, WI USA; 6 Burnett School of Medicine, Texas Christian University, Fort Worth, TX USA

**Keywords:** Leadership, Integrated curriculum, Longitudinal courses, Course directors, Health professions education, Generalist, LILIC

## Abstract

Recent shifts in health professions education have prompted significant curriculum reforms toward multidisciplinary, longitudinally integrated courses during the pre-clinical and clinical years of medical school curricula. Faculty who are accustomed to managing discipline-specific courses must now be trained to meet the challenges of implementing new integrated courses. Skills and competencies for Leaders in Longitudinal Integrated Curricula (LILIC) must be identified for faculty development efforts and to assist administrators in recognizing the attributes of well-qualified course/curriculum directors for their appointment/hiring decisions. Drawing on the collective expertise of faculty from multiple medical schools, we developed a framework to meet this new need. Herein, we present a shared mental model, a framework, and higher-level attributes important for the development of these curricular leaders. A proposed development process and future directions for this framework are also presented, underscoring its potential impact on faculty development and the broader educational landscape in health professions.

## Introduction

Many medical schools have undergone curriculum revision to integrate clinical science with multiple foundational science disciplines and, in doing so, have moved away from the 2 + 2 Flexnerian model of medical education [1]. In addition, it is now common to see programs implementing longitudinal courses in both the pre-clinical and the clinical years. These curriculum reform efforts aim to create more integrated curricula aligned with learner’s developmental stages that better reflect the interconnected nature of medical knowledge and practice [2]. Faculty skills needed to oversee these types of courses differ from those needed for traditional discipline-specific courses as they typically require coordinating more faculty across a breadth of disciplines and specialty areas. These curricular changes have also led to changes in administrative structures such that some schools now hire clinical, basic science, and health humanities faculty who are placed in integrated departments such as foundational sciences or medical education. Such structural adjustments necessitate a reevaluation of the skills and competencies required for effective medical education that we designate as Leaders in Longitudinal Integrated Curricula, which we abbreviated with LILIC.

LILIC is not to be confused with Longitudinal Integrated Clerkships, which were introduced to accelerate the move away from specialty-specific training to one that promotes patient-student relationships for students in their clerkship experiences [3]. The framework for skills and corresponding faculty development for those who lead longitudinal integrated clerkship are distinct with their focus on unique relational learning outcomes and are outside the scope of this monograph [4].

Recognizing the importance of leadership development for faculty, a few attempts have been made to identify the general attributes important for leaders in medical education [5–7], and more recently, there have been several attempts to identify competencies important for clinical educators [8–10]. However, most of those frameworks address broad leadership skills that are generally applicable to various educational practices [10–12], rather than the adaptation of the unique leadership skills necessary for the integrated and longitudinal nature of medical curricula. Moreover, at the individual institution level, most of the faculty development and training in medical schools focuses on procedural educational skill development, like objective setting, content development, multiple-choice assessment writing, and discrete teaching approaches targeted to the institution’s curricular needs.

In addition to the identification of general leadership qualities for medical educators, there have been several attempts to develop a central framework for educator excellence. For example, Harden et al. have proposed 12 roles of effective medical educators that include the teacher as a facilitator, role model, planner, and assessor among others [13], and subsequently defined the competencies and attributes expected of an effective teacher in three groups of technical competencies, approaches to teaching, and professionalism [14]. Broad teaching competencies (knowledge, skills, and attitudes) have been identified for those teaching in the clinical arenas as well [15]. Furthermore, 9 foundational entrustable professional activities (EPAs) for educators in the health professions were proposed in 2022 [16], but none of these frameworks for teaching excellence includes leadership skills for integrated medical curricula. In fact, one EPA for “designing and developing a course” states that, “Entrustment is limited to (re)design and (re)development of a course about one’s own or related area of expertise,” acknowledging that leadership in integrated courses requires a unique skill set that has not yet been defined in the literature [16]. These new teaching environments require a leadership framework or adaptation of existing frameworks to a specific context of integrated longitudinal courses that reflect the appropriate requirements for the faculty who lead them.

Frameworks in medical education are important as they provide the guiding principles and the structure in which to consider them. Two common frameworks include the Learner Assessment Framework [17] and the Competency-Based Framework [18], both of which are useful in current medical curricula and help guide curriculum development and assessment practices, respectively. Here, we propose the framework based on the generalist leadership principle that (1) provides clarity for faculty who wish to become leaders of longitudinal integrated curricula, (2) facilitates consistency for those who train them, (3) improves the evaluation of these education leaders, and, most importantly, (4) drives improvement of the educational learning environment in medical education.

## Process

Faculty from three medical schools who responded to a post on the DR-ED listserv (October 13, 2022) met at a national medical education conference (The Generalists in Medical Education, Annual Meeting, Nashville, TN) in 2022. After discovering that there was a gap in the literature on developing leaders who oversee longitudinal or integrated medical curricula, they affirmed the need for a framework and a shared mental model for assisting in the development of these curricula leaders. After the conference, the participants formed the “LILIC” consortium to address the identified gap. Since then, the consortium has grown to eight members from five medical schools, and each of the faculty members has extensive experience of leading integrated curricular and currently holds curricular leadership and/or faculty development roles in their respective institutions. The group conducted monthly meetings where:

- The need for leadership specific to the context of a longitudinal, integrated curriculum was defined and articulated;
- A literature search was conducted by individual members using shared key search terms around leadership theories and frameworks and various adaptations in medical education;
- Literature review findings were analyzed in monthly meetings, and further identification of the gap was conducted, which led to the shared research goal to develop a framework for leadership in the specific context;
- A shared mental model for LILIC was drafted detailing curricular- and systems-level considerations as well as the statements of outcomes for effective LILIC;
- An overarching LILIC framework based on “generalist” principles was created and the identification of the relevant competencies was started;
- A practical tool for faculty development and self-assessment was proposed in the interim to include a selection of high-level leadership attributes and management skills required by distinct tasks associated with longitudinal and integrated curriculum in three broad categories (intrapersonal, interpersonal, and organizational/system);
- The initial work to establish a shared mental model for LILIC was presented as a poster at the 2023 Annual International Association of Medical Science Educators (IAMSE) in Cancun, Mexico [19].

## Results

**Fig. 1:**
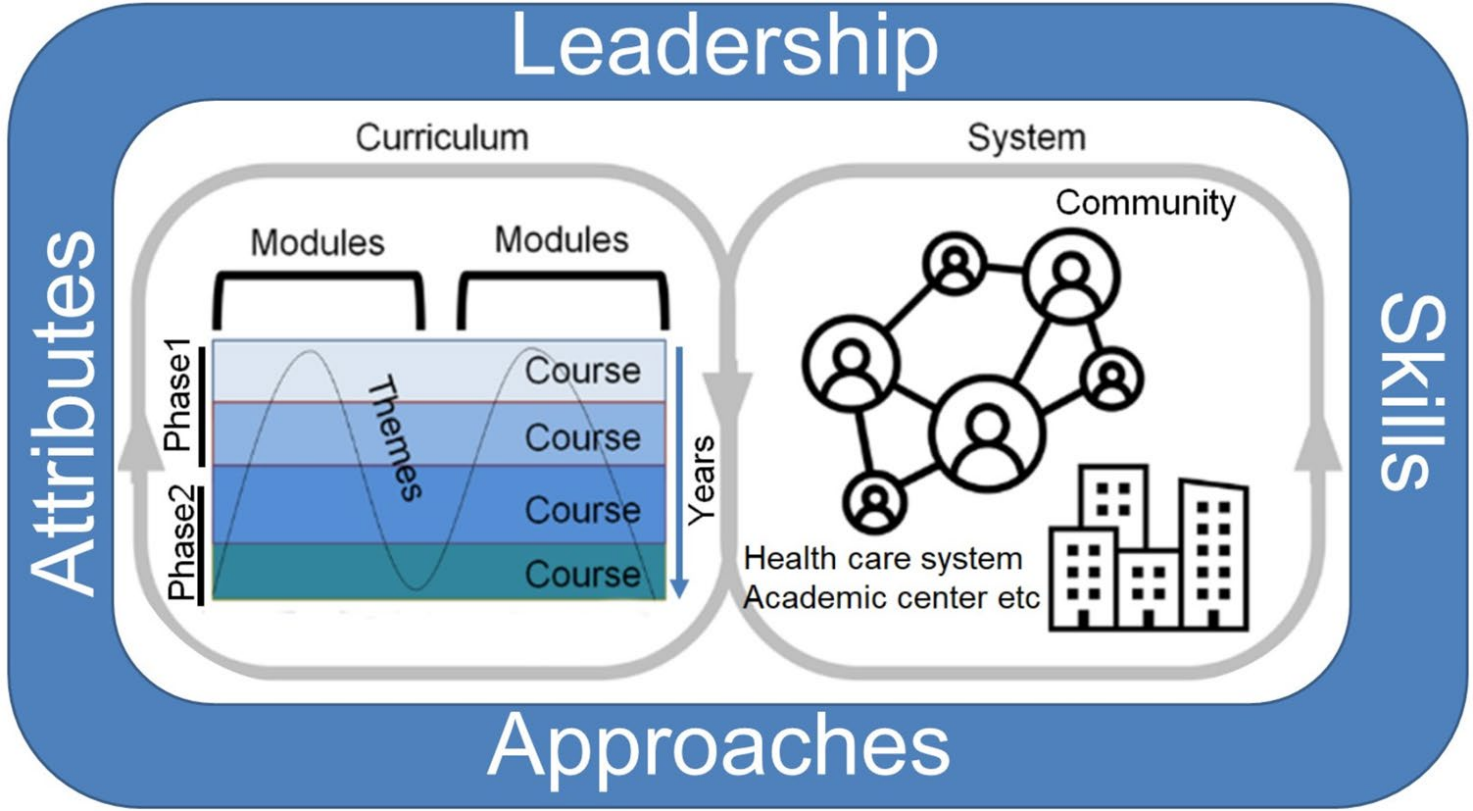
Illustration of a shared mental model depicting leadership in a longitudinal integrated curriculum (LILIC) and its components. This model helps visualize the complex interconnected nature of integrated curricula to inform leadership competencies. Medical curricula may be composed of multiple courses where a single course is typically offered within a distinct phase of students’ development or modules that are typically instructional units within a course that can be organized by topics. Themes are learning experiences organized around a unifying idea that is repeated or weaved, as depicted, through multiple modules, courses, or different phases of student development, involving consideration for all horizontal, vertical, and longitudinal aspects in its implementation. The gray line represents horizontal (within a phase of the curriculum), vertical (across phases, such as pre-clinical or clinical), and longitudinal (throughout the duration of the educational program) elements of the curriculum, illustrating the progression of time and increasing complexity of learning. These elements interact with larger systems such as the healthcare system (depicted as a building) and community (depicted as circles of people connected with one another). The blue outer box represents leadership that addresses both curriculum and connected systems of healthcare and community. Leadership includes personal attributes, skills, and broader approaches, all of which must adapt to the integrated and interconnected nature of the curriculum and its larger system

In order to come to an agreement on the nature of the curriculum and environment that we operate in, we first developed a shared mental model that includes the common modern medical education curriculum features as well as the general approaches for dealing with this curriculum in the academic center, healthcare system, or community (Fig. 1). Shared mental models have been shown to help teams make better decisions and have been used widely in medical education [20]. The components of the model include the following characteristics, often found in modules in medical education curriculum: (1) integrated horizontally across different subjects such as different basic science disciplines and clinical science in a distinct phase and vertically across different phases of medical education, (2) longitudinal throughout the entire education program, and (3) thematic with patient-centered or system/societal need-based themes interleaved through discrete courses and modules. The recognition of the intimate relationship between the curriculum and the system in which it operates (e.g., medical school, hospital or university, and community) is also depicted. For example, the pre-clerkship curriculum may be composed of various courses that integrate multiple disciplines, clinical skills, and health science citizenship, as well as a community service program that incorporates social justice and advocacy. The learning experiences may occur in various physical locations and contexts, and curriculum outcomes may be measured in the learner’s knowledge and attitude in a clerkship phase. The model suggests that, to lead such curricula, faculty should possess certain *attributes* (defined as inherent qualities or characteristics), acquire *skills* (defined as abilities that can be learned and refined), and develop *approaches* (defined as global strategies to accomplish goals), targeted to interconnected aspects of the curriculum itself and also with the community and system to meet the curricular needs. Further, the model highlights that enhanced collaboration across diverse expertise in various settings is needed.

As we began to construct a framework for faculty who lead integrated and longitudinal curricula, we considered the importance of the “generalist mindset.” The generalist versus specialist paradigm was introduced by Freeman Dyson, where he divided mathematicians into subgroups of “birds and frogs” to describe those with a broader view versus those who have an in-depth perspective, both of which are needed to tackle the world of mathematics that is both broad and deep [21]. Subsequently, the benefits of a generalist made popular in the book Range by David Epstein include resilience, which is needed in today’s complex and ever-changing world [22]. This is to be differentiated from the term generalist in the practice of medicine, which describes the need and benefit of generalist physicians to focus on whole-person care through a transdisciplinary approach [23–25]. The common understanding drawn from these published works is that specialists have specific disciplinary-based knowledge; in contrast, generalists have a broad understanding across several disciplines. Decreased focus on specialization may also increase creativity and adaptability, allowing the individual to make stronger connections across disciplines and engage more readily in critical thinking, both of which are very important when collaborating with specialists to direct multidisciplinary courses.

To select a leadership framework targeted for the integrated curricula, we first conducted a literature review on leadership theories and models, such as change or transformational leadership, and faculty development to promote leadership in medical education. We analyzed the target population, context, and outcomes of leadership reported and how reported leadership would be applied to faculty teaching in longitudinal integrated curriculum (not longitudinal integrated clerkships). We categorized attributes and skills, as well as outcomes mentioned, into categories of intrapersonal, interpersonal, and organizational/system levels. Simultaneously, we worked on defining outcomes of effective leadership in a longitudinal integrated curriculum in the same four categories, such as faculty empowerment in understanding the scope of responsibilities and system-level goals that leaders who understand the value added to the system and community. We next developed a list of unique tasks and characteristics of a longitudinal integrated curriculum that leaders must address to produce the desired outcomes. Then, we tried to match the leadership framework and its components to the tasks and outcomes specific to the longitudinal integrated curriculum. This synthesis exercise revealed that specific elements of such a curriculum require a unique combination of attributes and skills and that existing models cannot be applied to address them effectively. Individual skills and attributes might be common to other leadership models, but small subsets or combinations were critical because of the interconnected challenges of the longitudinal integrated curriculum. The importance of placing leadership in a specific context has been emphasized to ensure successful outcomes of leadership training programs and to facilitate meaningful changes [12]. Taken together, we determined that we needed to construct a new leadership framework that clearly defines the specific longitudinal integrated curriculum context as a key component and provides a broad interpretation of the roles and responsibilities of leadership within that context.

**Fig. 2:**
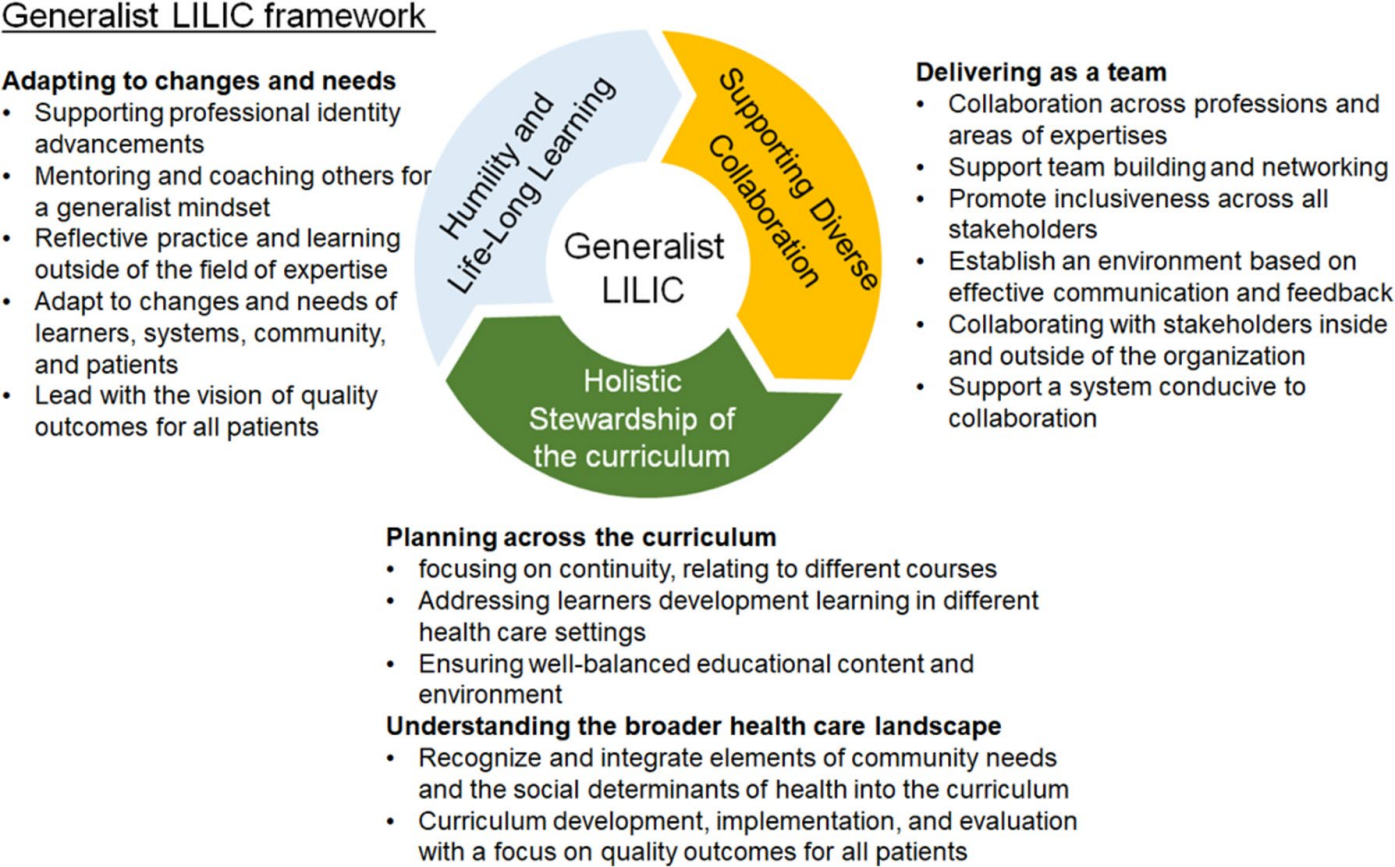
The Generalist LILIC Framework: A Generalist Approach to Leadership in Longitudinal Integrated Curricula. This figure illustrates the Generalist LILIC (Longitudinal Integrated Leadership in Curriculum) framework, which adopts a generalist approach as its overarching principle and consists of three interconnected competency domains depicted as interlocked parts in a circle. This generalist approach encourages leaders to develop broad, adaptable competencies across all spheres of leadership corresponding to three domains. The three competency domains address unique challenges due to the complex and connected nature of longitudinal integrated curricula. Examples of such challenges are listed next to the domain: (1) Humility and Life-Long Learning (Intrapersonal): Focuses on adapting to changes, mentoring for a generalist mindset, and leading with a vision of quality patient outcomes. (2) Supporting Diverse Collaboration (Interpersonal): Emphasizes team delivery, cross-professional collaboration, and establishing effective communication environments. (3) Holistic Stewardship of the Curriculum (Organizational/System): Encompasses curriculum planning, ensuring continuity between courses and phases, and integrating community needs and system outcomes into the curriculum. While specific challenges are listed under each domain, addressing real-world issues often requires combinations of competencies, enabling leaders to navigate the complexities of modern health profession education effectively

Applying the tenets of a generalist, we propose a framework that emphasizes the “generalist leader” and includes skills needed to manage a longitudinal integrated curriculum. We define the generalist leader as a leadership style characterized by the synthesis and adoption of a broad range of knowledge, perspectives, skills, and attitudes to effectively navigate complex and interconnected problems. The three major domains for a generalist leader in a longitudinal integrated curriculum, as shown in Fig. 2, include (1) humility and life-long learning governing the intrapersonal sphere, (2) supporting diverse collaboration governing the interpersonal sphere, and (3) a holistic understanding of the curriculum governing the system/community sphere. Organizing the leadership into three domains in three different spheres is consistent with the established theory that leadership is to lead self, others, and organization/system [26] and aligned with three levels of the attributes and skills as described earlier. Examples of the complex and interconnected problems of leading such a curriculum, necessitating the skills, attitudes, and knowledge under each domain, are shown in Fig. 2.

Humility and life-long learning specify a provisional attitude toward knowledge in that knowledge is alive and in flux, requiring ongoing learning, self-assessment, and solicitation of feedback for continuous improvement to adapt to the changing needs of learners, systems, and community. Competency in this domain is required for educators to learn broadly and judge when/who to consult for expertise. It is aligned well with the principles of the growth mindset [27]. It is also important to lead with the vision of quality outcomes that are broadly defined and complex, including the outcomes for all patients and communities.

Supporting diverse collaboration specifies competencies to support the active and inclusive engagement of individuals from various backgrounds, professions, and perspectives while working toward the shared goals of delivering a cohesive curriculum. It refers to the ability to not only collaborate across, but also advocate for processes that are conducive to such inclusive participation.

A holistic understanding of curriculum specifies competencies related to directing complex integrated curricula through planning, organization, and implementation with a keen awareness toward larger system goals of improving patients and community health outcomes and the overall vision. Competencies in this domain are required to ensure that the medical education curriculum understands and incorporates the broader healthcare landscape and community needs that improve social justice.

As an illustration of how the three domains work together, leadership in curricular renewal to integrate basic and clinical science can be supported through the following key components: (1) the holistic understanding of the curriculum domain emphasizes the leader’s ability to see connections across disciplines and phases of education for effective integration throughout the curriculum and connect curricular outcomes to system outcomes; (2) the supporting diverse collaboration domain highlights the importance of facilitating collaboration between clinician and foundational science faculty in designing optimal learning experiences and assessments that are longitudinal in nature starting during the pre-clinical phase and revisited and built-upon in clinical years; and (3) the humility and life-long learning domain acknowledges that faculty must recognize the need to operate largely outside of their discipline/specialty areas in their leadership roles and be willing to acquire a broader perspective, in order to continually adapt to new educational approaches, clinical practices, and healthcare system goals.

Development of competence in all these domains is beneficial for a future or current leader of the integrated and or longitudinal curriculum. We are currently developing sub-competencies, including descriptions and levels under each domain, but these are beyond the scope of this report. To date, the proposed generalist leadership framework, including three competency domains reported here and provisional sub-competencies, has been presented at three regional and national medical education conference workshops to engage experts and stakeholders. Although the competency framework is provisional at this time, preliminary analysis of participants’ anonymous responses indicated greater than 85% overall agreement for three competency domains as proposed to be relevant for LILIC.

In facilitating the practical application of the generalist LILIC framework to faculty development, while continuing to develop full competency tools, we sought to further specify some specific attributes and skills relevant to LILIC. We reviewed various leadership skills and attributes from the health science education literature (for example, van Diggle et al. and Lieff and Albert [7, 28]) and prioritized them based on specific tasks and challenges associated with LILIC and consortium members’ experience of leading such curricula. The detailed literature review and analysis process was described earlier. These attributes and skills are listed in Table 1. Through iterative discussion, the attributes and skills were grouped into three different levels: intrapersonal, interpersonal, and organizational/system. We initially clustered attributes and skills into four themes of practice, intrapersonal, interpersonal, organization, and systemic, similar to the approach by Lieff and Albert [28]. However, we felt that tasks and corresponding skills required for organizational tasks in creating a shared vision and systemic tasks in strategic navigation largely overlap. Thus, we grouped organizational and system-related attributes and skills together into a single level. This resulted in grouping skills and attributes to three levels — intrapersonal, interpersonal, and organizational/system that are aligned with the three competency domains of the generalist LILIC framework as depicted in Fig. 2 (Humility and Life-Long Learning, Supporting Diverse Collaboration, Holistic Stewardship of the Curriculum governing the intrapersonal, interpersonal, and systemic spheres respectively). Lastly, we matched each attribute with relevant leadership skills, as well as management-related skills. This was done to depict what skills and innate personal qualities align to form a competency, which consists of knowledge, attitude, and skills, while recognizing that there is a managerial or technical aspect of “doing things” benefiting leadership. These attributes and skills are designed to be broad and encompass some of the important aspects of the generalist mindset of the medical educator as a leader and practitioner.

**Table 1:** Attributes, leadership skills, and management skills for Leaders in Longitudinal Integrated Curricula (LILIC)

Levels	Attributes	Leadership skills	Management skills
Intrapersonal	Visionary	Goal setting	Establish agenda, policy making
	Honest and integrity	Continuous quality improvement; engages in self-reflection and learning about personal biases	
	Persistence despite challenges	Time management, conflict resolution	
	Self-control	Time management, conflict resolution	Manage own expectations and time
	Adaptability	Problem-solving; change management	
Interpersonal	Social perceptiveness	Goal setting; conflict resolution	Delegation; feedback collection
	Humility	Active listening; seeking feedback	
	Emotional intelligence	Conflict resolution; communication	
	Collaborative	Communication with stakeholders; explaining decisions; developing and maintaining diverse teams	Schedule meetings, maintain structure, staffing/personnel monitor
	Responsibility to others	Conflict resolution; developing and maintaining team; mentoring and advising; eliminating barriers to performance	Feedback collection, conflict recognition system, placing reward system
Organizational/system	Outcome focused	Goal, objective, plan setting	Outcome collection mechanism; review product on time
	System-based/holistic thinking	Analytic and situational; considering the overall systems and its individual parts (awareness of interconnectedness); promotes equity and inclusion	Mapping and feedback loop analysis
	Strategic thinking/Critical thinking	Analytic and plan development; risk taking	Analysis, performance metric collection risk assessment,
	Developmental thinking/Growth mindset	Learner-specific stage setting; individualization based on growth	Milestone-based assessment Progress monitoring based on multiple indices

This table outlines key attributes, leadership skills, and management skills that are particularly relevant for leaders of integrated and longitudinal curricula in medical education. These elements were identified through a literature review of leadership and prioritized based on the specific tasks and challenges associated with LILIC. The attributes and skills are organized into three levels (intrapersonal, interpersonal, and organization/systemic) that are aligned with the domains of the generalist LILIC framework. Each row presents an attribute along with corresponding leadership and management skills, illustrating how innate qualities can be complemented by learnable skills to form comprehensive competencies. This table serves as a practical tool for faculty self-assessment and training in faculty development in leading integrated medical curricula

## Application

The generalist leadership framework that we propose can be applied to any faculty who is tasked or wishes to lead courses, modules, or theme-based programs in a longitudinal integrated curriculum. Most faculty step into those leadership roles without training on global approaches or required skill sets. By understanding themselves as a generalist leader, those faculty now have a way to articulate their leadership responsibilities across broad curricular issues. They can also use the framework as a self-development tool to view their existing attributes as a strength while seeking to develop skills that they do not have to enhance their leadership effectiveness. The framework can also be used for administrators or department leadership who need to assign faculty to lead integrated longitudinal courses or programs, evaluate readiness or effectiveness, and/or provide the faculty with prioritized training to develop the necessary skills.

In a hypothetical scenario, consider Dr. Smith, PhD who teaches biochemistry and facilitates case-based learning in the integrated pre-clerkship curriculum. He is motivated to become a course director for longitudinal integrated foundational science course, which includes both clinical science principles and multiple disciplines around three organ systems in the pre-clerkship phase. In preparation for becoming the course director, he performs a self-evaluation and discovers that he has strength in the “humility and lifelong learning” domain while recognizing that he is not equipped for tasks associated with “holistic understanding of the curriculum” domain, especially lacking the understanding of how the course is connected to the whole curriculum and system outcomes. After the self-evaluation, he approaches the department chair, asks to be a part of the curriculum committee, and attends various seminars related to situational system-based decision-making.

As another scenario, consider Dr. Allen, who is the chair of the medical education department and needs to assign a director to oversee the pre-clinical skill course that embeds health system science and medical humanism themes. She has a faculty clinician who is an endocrinologist in mind, as a candidate. In reviewing the faculty’s CV and using the generalist framework, she recognizes that this individual has a strong vision for patient and community outcomes but lacks experience in supporting diverse collaborative teams. In a meeting with the faculty, Dr. Allen describes how the position requires a broad understanding of various educational content, not having to be the expert in each, and why the faculty member’s vision would be a strength to lean on. She clarifies this with various stakeholders, and she offers support to the faculty in seeking faculty development in interpersonal skills and shared goal setting.

These examples highlight how the framework can be used by faculty leaders and those who identify and support them to facilitate the articulation of responsibilities and the attributes and skills needed to lead the integrated longitudinal curricula.

## Conclusion

Leadership development in the healthcare educational space has taken on greater importance as we implement complex interdisciplinary and longitudinal courses. Skills and attributes, as well as frameworks, exist for health professions education on a broader level. However, in order to help identify, train, and develop faculty who will productively lead and manage these courses, a more targeted framework was developed. Using the generalist mindset as a starting point, we embarked on developing a shared mental model for the curriculum environment of today, as well as a framework to help establish some guiding principles and an agreed-upon template to start building meaningful competencies. We then described a set of skills and attributes that we felt were essential for faculty leading these types of courses and that were consistent with a generalist approach.

To facilitate practical implementation of the framework and the proposed competencies, we have begun developing a self-assessment tool that encompasses each domain and its attributes. We have presented the framework and the tool at conferences in the form of workshops so that we can gauge interest in adoption by other experts and practitioners and for further refinement. We are in the early phase of collecting and analyzing feedback on the domains and provisional sub-competencies from appropriate stakeholders.

Other competency frameworks, such as the Clinical Educator Milestones developed by the ACGME/AAMC [10], took years to prepare and adopt, and we expect a similar development trajectory. However, it is important to discuss how we anticipate this framework and evaluation tools will be used and by whom; once fully developed and generally accepted by various stakeholders, (1) we expect this tool will help faculty in medical schools in self-assessment and advocating for support as they take on curricular change moving away from discipline-focused pre-clerkship education and/or implementing longitudinal curricula. (2) We also expect that this framework and tool will be valuable for senior leadership when looking for faculty or teams to lead these courses, articulating responsibilities, and conducting formative evaluations to improve performance. (3) Finally, these can be used as part of faculty and professional development training to target specific skills and approaches, alongside educational procedural skill training.

## Data Availability

The information in this monograph is not data based/centric and thus, it is not applicable to make a statment to that affect.
